# Insulin and IGF-1, but not 17β-estradiol, alter the subcellular localization of MIER1α in MCF7 breast carcinoma cells

**DOI:** 10.1186/s13104-015-1336-0

**Published:** 2015-08-18

**Authors:** Shengnan Li, Gary D. Paterno, Laura L. Gillespie

**Affiliations:** Terry Fox Cancer Research Laboratories, Division of BioMedical Sciences, Faculty of Medicine, Memorial University of Newfoundland, St John’s, NL A1B 3V6 Canada

**Keywords:** MIER1α, Transcriptional regulator, Breast cancer, MCF7, Insulin, IGF-1, Estrogen, Subcellular localization

## Abstract

**Background:**

MIER1α is a transcriptional regulator that interacts with estrogen receptor α and inhibits estrogen-stimulated growth of breast carcinoma cells. Interestingly, analysis of MIER1α subcellular localization in breast samples revealed a stepwise shift from the nucleus to the cytoplasm during progression to invasive carcinoma. Previously, we demonstrated that MIER1α is nuclear in MCF7 cells yet it does not contain a nuclear localization signal. Instead MIER1α is targeted to the nucleus through interaction and co-transport with HDAC 1 and 2.

**Results:**

In this study, we demonstrate that treatment of MCF7 breast carcinoma cells with either insulin or insulin-like growth factor affects the subcellular localization of MIER1α. Both factors reduce the percentage of cells with nuclear MIER1α from 81 and 89 to 41 and 56 %, respectively. Treatment with 17β-estradiol, on the other hand, had no effect and MIER1α remained nuclear.

**Conclusions:**

Our data demonstrate that insulin and IGF-1 can contribute to loss of nuclear MIER1α in the MCF7 breast carcinoma cell line.

## Background

MIER1α is a transcriptional repressor [1, 2] that has been implicated as a tumour suppressor in breast cancer [3]. It interacts with ERα and inhibits estrogen-stimulated anchorage-dependent growth of breast carcinoma cells [3]. Moreover, analysis of patient breast biopsies revealed a dramatic reduction in nuclear MIER1α during progression, from 75 % nuclear MIER1α in normal samples to 51 % nuclear in ductal carcinoma in situ to 4 % nuclear in invasive ductal carcinoma [3]. Thus loss of nuclear MIER1α is associated with breast cancer progression.

MIER1 represses transcription through several distinct mechanisms: it can recruit histone deacetylase (HDAC) 1 and 2 to the promoter of responsive genes [1]; it can bind Creb binding protein (CBP) and inhibit its histone acetyltransferase activity [4]; finally, it can interact directly with transcription factors such as Sp1 and displace them from their cognate site on target gene promoters [2]. All of these functions are dependent on localization of MIER1α in the nucleus, yet it does not contain a functional NLS [5]. Instead, translocation into the nucleus is dependent on interaction and co-transport with HDAC1 and 2 [6]. In this report, we show that localization of MIER1α in the nucleus of MCF7 cells is significantly reduced by treatment with insulin or IGF-1, but not by 17β-estradiol (E2). This suggests that insulin or IGF-1 could attenuate MIER1α’s transcriptional repressor/chromatin modifying functions in MCF7.

## Methods

The MCF7 breast carcinoma cell line was obtained from the ATCC and cultured in DMEM (GIBCO) containing 10 % serum [7.5 % calf serum (CS) + 2.5 % fetal bovine serum (FBS)] (GIBCO), in a humidified 37 °C incubator with 5 % CO_2_. Insulin was purchased from Life Technologies and used at a concentration of 10 ug/ml. IGF-1 was purchased from PeproTech and used at a concentration of 10 ng/ml. 17β-estradiol was purchased from Sigma-Aldrich and used at a concentration of 10^−8^ M. For experiments using 17β-estradiol, cells were cultured in phenol red-free DMEM (GIBCO) supplemented with 10 % charcoal-stripped FBS (Hyclone). Cells were treated for 4 h with insulin, IGF-1 or 17β-estradiol prior to fixation. Construction of the human *mier1α* sequence (GenBank: AY124188) in the CS3 + MT vector has been described previously [1]. Transient transfection, confocal microscopy, antibodies used and Z-stack analysis were performed as described in [6, 7]. Subcellular localization was scored as ‘nuclear’ if the nucleus was intensely stained, with little or no cytoplasmic staining; ‘cytoplasmic’ if staining was primarily in the cytoplasm, with little or no staining in the nucleus; ‘whole cell’ if both the nucleus and cytoplasm were stained [6]. Statistical analysis was performed using a two-sided Fisher’s exact test.

## Results and discussion

### Insulin alters nuclear localization of MIER1α in MCF7 cells

**Fig. 1:**
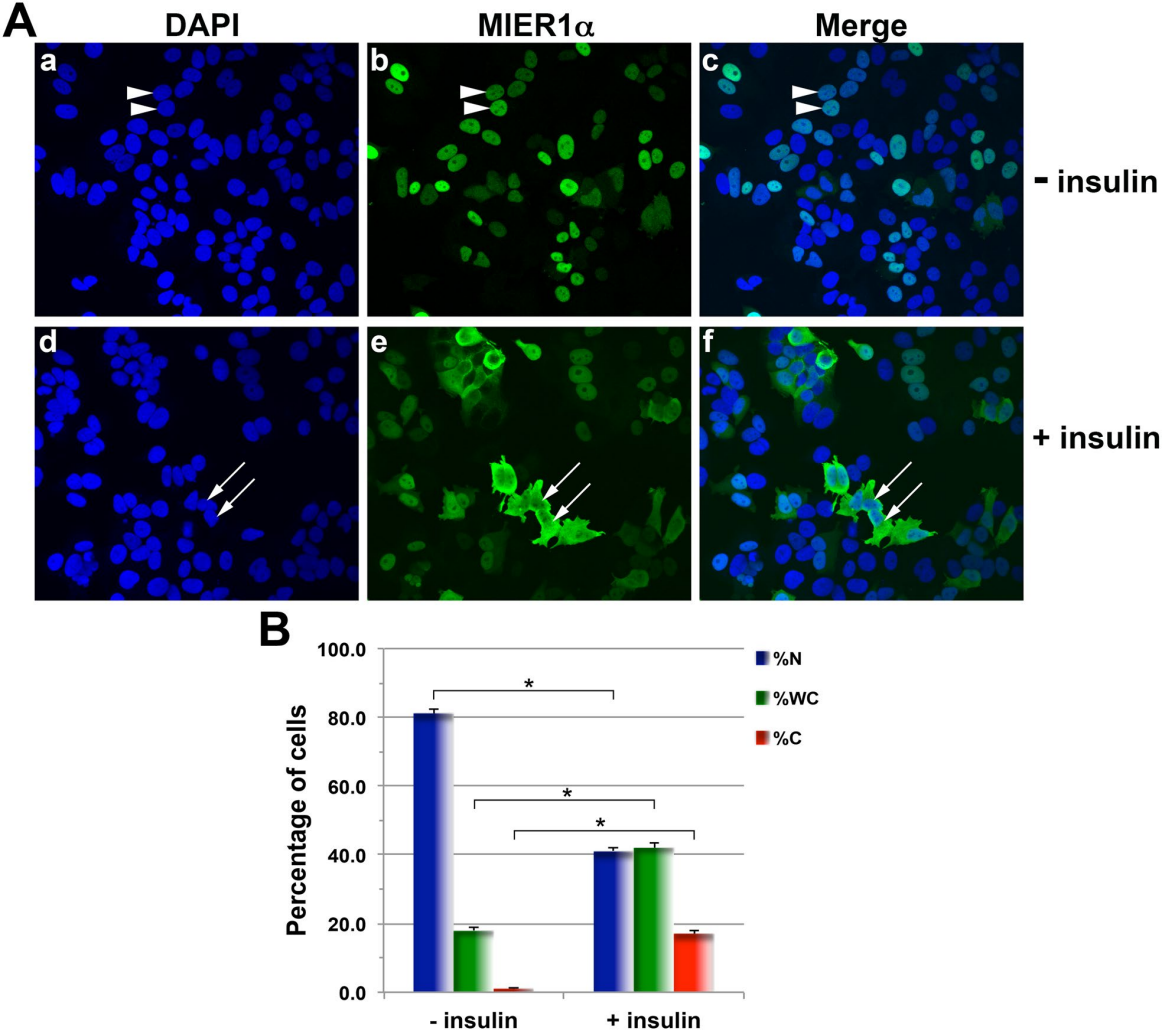
Insulin treatment reduces nuclear localization of MIER1α. MCF7 cells were transfected with myc-tagged *mier1α* and treated 24 h later with either vehicle (*panels* **a**–**c**) or 10 ug/ml insulin (*panels* **d**–**f**). Cells were fixed 4 h after the addition of insulin and localization was analyzed by confocal microscopy. Random fields were selected and the staining pattern of each cell within the field was scored as ‘nuclear’, ‘whole cell’ or ‘cytoplasmic’, using sequential Z-stage scanning. **A** Shown are Z-stacks compiled into individual images, illustrating stained nuclei (DAPI; *panels* **a**, **d**), MIER1α localization (9E10 anti-myc tag antibody and an AlexaFluor-488 secondary antibody; *panels* **b**, **e**) and merged channels (*panels* **c,** **f**); arrowheads show examples of nuclear staining and *arrows* show whole cell staining. **B** Histogram showing the results of 3 independent experiments; the MIER1α localization pattern of >1000 cells was scored. Plotted is the percentage of cells in each category ±SD; *Asterisk* indicates that the difference was statistically significant, *p* < 0.05 (Fisher’s exact test)

We have shown previously that MIER1α is targeted to the nucleus in MCF7 cells despite the lack of an intrinsic NLS [6, 7]. In those studies, cells were cultured in DMEM containing 10 % CS/FBS. Several laboratories, including the ATCC, add 10 ug/ml insulin to culture media for MCF7 cells, however when we added insulin, we noticed a change in the subcellular localization pattern of MIER1α. To investigate this effect more thoroughly, we analysed *mier1α*-transfected MCF7 cells by confocal microscopy. In the presence of insulin, only 41 % of cells had exclusively nuclear MIER1α (Fig. 1Ad–f, B), compared to 81 % of cells in the absence of insulin (Fig. 1Aa–c, B). The percentage cells with MIER1α in both the nucleus and cytoplasm (whole cell staining) increased in the presence of insulin, from 18 to 42 % (Fig. 1B). Likewise, the proportion of cells with exclusively cytoplasmic MIER1α increased over tenfold, from 1 to 17 % (Fig. 1B). These results demonstrate that in the presence of insulin, localization of MIER1α in MCF7 cells is shifted from the nucleus to the cytoplasm.

**Fig. 2:**
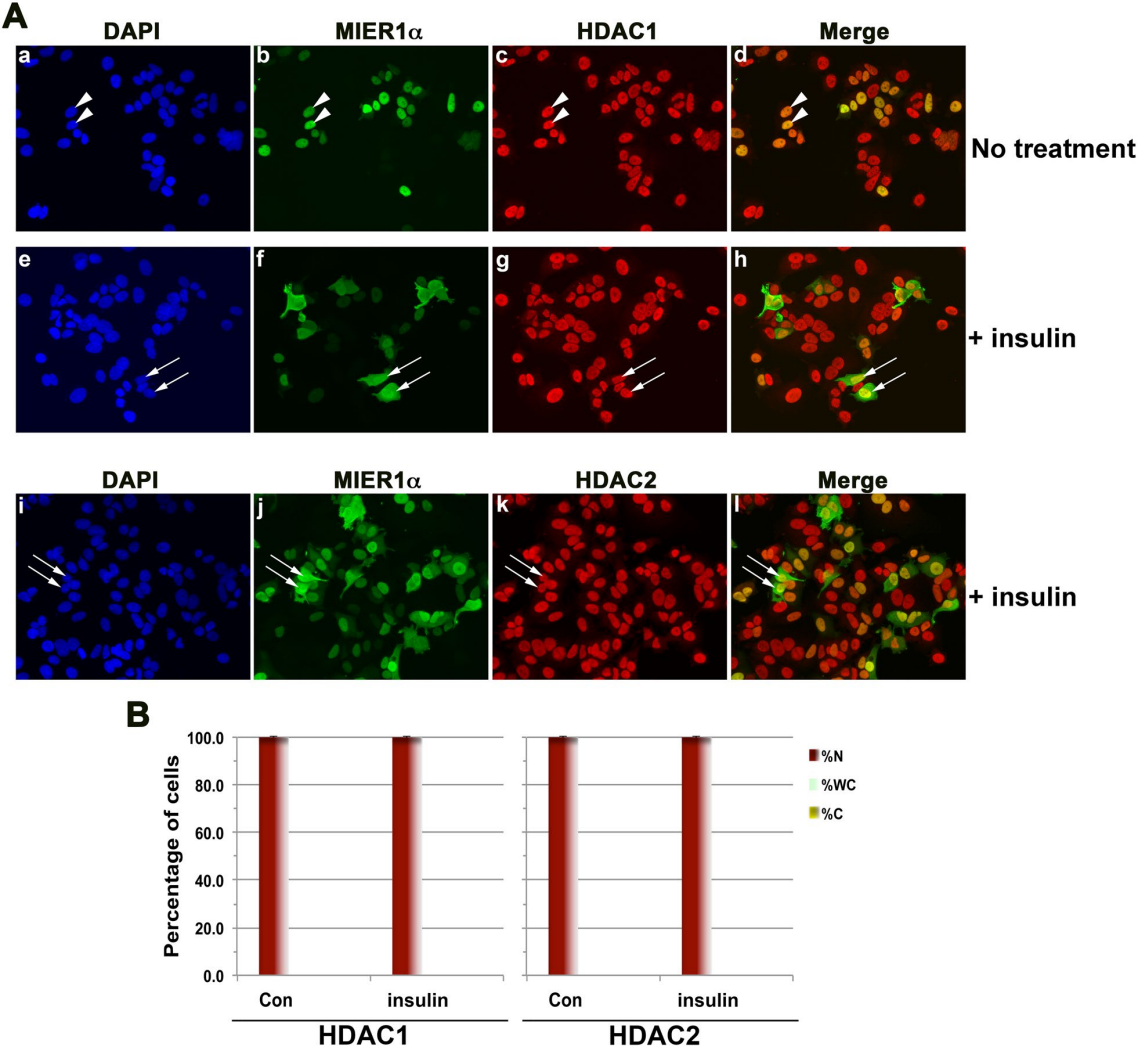
HDAC1 and 2 localization are not affected by insulin. Cells were transfected, treated with insulin and prepared for confocal microscopy as described in Fig. 1. **A** Compiled Z-stacks showing nuclei (DAPI; *panels* **a**, **e**, **i**), MIER1α (AlexaFluor-488, *panels* **b**, **f**, **j**), HDAC1 (AlexaFluor-647, *panels* **c**, **g**), HDAC2 (AlexaFluor-647, *panel* **k**) and merged 488 and 647 channels (*panels* **d**, **h,** **l**); *arrowheads* show examples of nuclei and *arrows* show whole cell staining. **B** Histogram showing the localization of HDAC1 and HDAC2 in untreated (Con) and in insulin treated MCF7 cells; plotted is the percentage of cells in each category ±SD from three independent experiments

Our previous research demonstrated that MIER1α localizes to the nucleus through interaction and co-transport with HDAC1/2. Therefore we investigated whether insulin also affected localization of HDAC1/2. Confocal analysis demonstrated that while insulin reduces nuclear accumulation of MIER1α (Fig. 2Ab, f, j), it does not affect localization of HDAC1 or 2 (Fig. 2Ac, g, k) and both were 100 % nuclear (Fig. 2B).

### Nuclear accumulation of MIER1α is affected by IGF-1, but not by E2

**Fig. 3:**
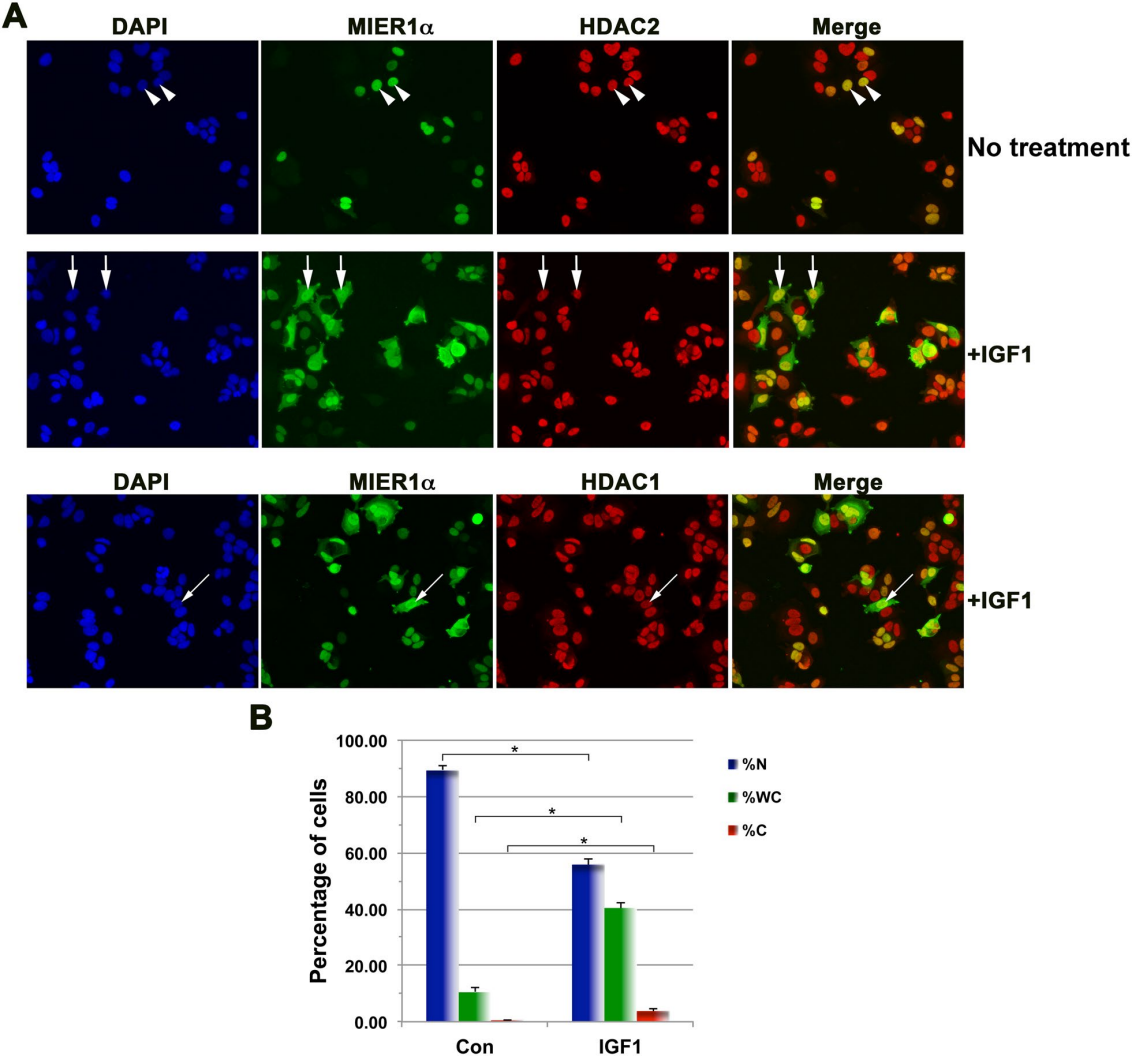
IGF-1 reduces nuclear localization of MIER1α. MCF7 cells were transfected with myc-tagged *mier1α* and treated 24 h later with either vehicle (*panels* **a**–**d**) or 10 ng/ml IGF-1 (*panels* **e**–**l**). Cells were fixed 4 h after the addition of IGF-1 and localization was analyzed by confocal microscopy, as in Fig. 1. **A** Compiled Z-stacks showing nuclei (DAPI; *panels* **a**, **e**, **i**), MIER1α (AlexaFluor-488, *panels* **b**, **f**, **j**), HDAC1 (AlexaFluor-647, *panels* **c**, **g**), HDAC2 (AlexaFluor-647, *panel* **k**) and merged 488 and 647 channels (*panels* **d**, **h,** **l**). **B** Histogram showing the results of two independent experiments; the MIER1α localization pattern of >1000 cells was scored. Plotted is the percentage of cells in each category ±SD; *Asterisk* indicates that the difference was statistically significant, *p* < 0.05 (Fisher’s exact test). Note that nuclear localization of HDAC1 and HDAC2 was unaffected by IGF-1

IGF-1 is closely related to insulin and both can interact with the insulin and IGF receptors, albeit with differing affinities [8]. In addition, there is a wealth of evidence implicating IGF-1 in breast cancer development and progression (reviewed in [9] ) and it has been shown to increase invasiveness of MCF7 cells [10]. Since MCF7 cells express receptors for both insulin and IGFs [11], we explored the possibility that IGF-1 also affects localization of MIER1α. As expected, confocal analysis demonstrated that IGF-1 had a similar effect on nuclear accumulation of MIER1α (Fig. 3Ab, f, j, B). IGF-1 reduced the percentage of cells with nuclear MIER1α from 89 to 56 % and increased the percentage with ‘whole cell’ staining from 10 to 40 %. The percent with ‘cytoplasmic’ MIER1α was also increased from 0.3 to 4 %. Thus, both insulin and IGF-1 have similar effects on the subcellular localization of MIER1α in MCF7.

**Fig. 4:**
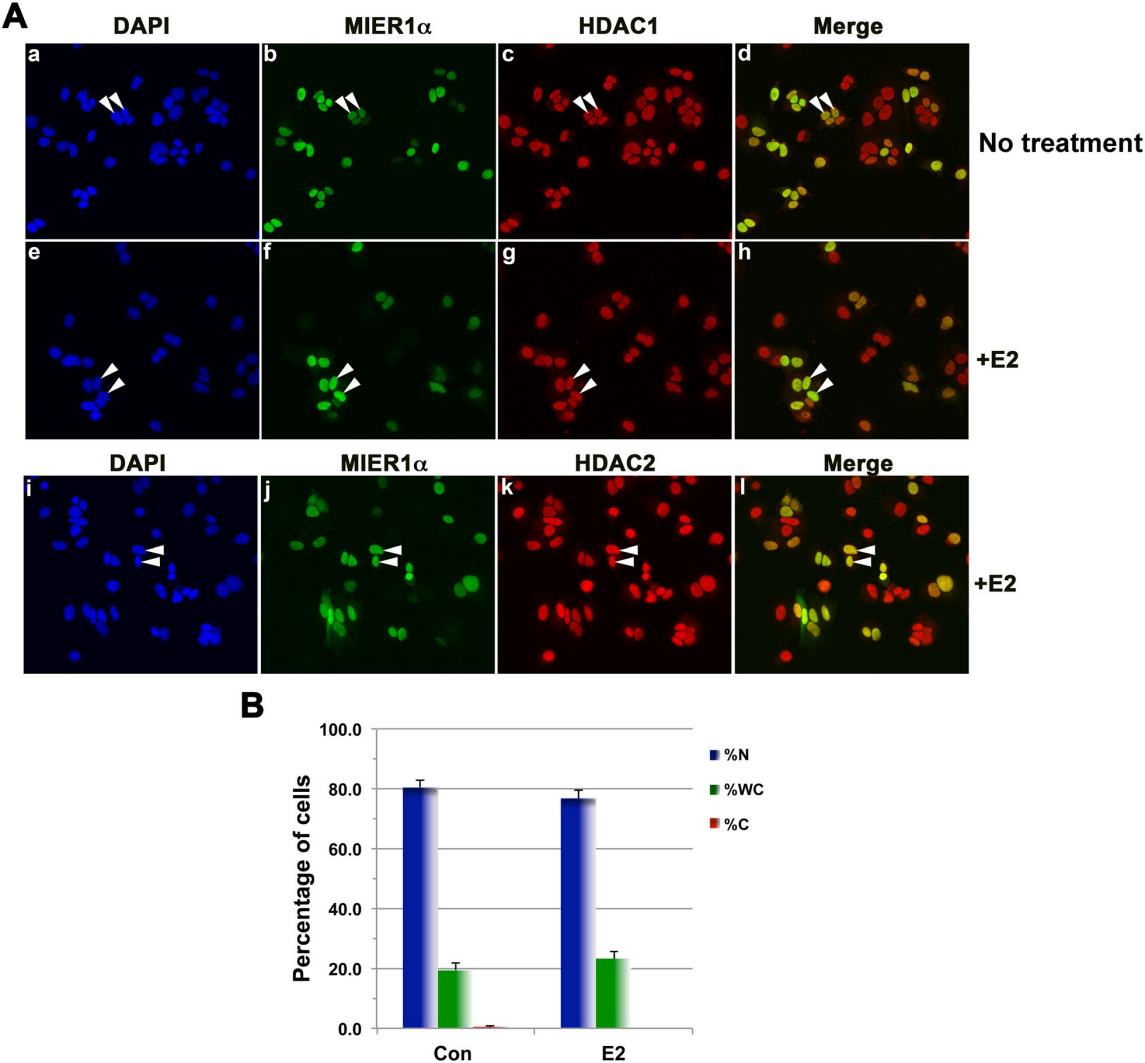
E2 has no effect on subcellular localization of MIER1α. MCF7 cells were transfected with myc-tagged *mier1α* and 24 h later, either vehicle (*panels* **a**–**d**) or 10^−8^M E2 (*panels* **e**–**l**) was added. Cells were fixed 4 h later and localization was analyzed by confocal microscopy, as in Fig. 1. **A** Compiled Z-stacks showing nuclei (DAPI; *panels* **a**, **e**, **i**), MIER1α (AlexaFluor-488, *panels* **b**, **f**, **j**), HDAC1 (AlexaFluor-647, *panels* **c**, **g**), HDAC2 (AlexaFluor-647, *panel* **k**) and merged 488 and 647 channels (*panels* **d**, **h**, **l**). **B** Histogram showing the results of two independent experiments; the MIER1α localization pattern of >500 cells was scored. Plotted is the percentage of cells in each category ±SD; there was no statistically significant difference between the percentage of control and E2 treated cells in each of the three categories, *p* > 0.05 (Fisher’s exact test)

Insulin and IGFs are potent mitogens for MCF7 cells [12], leading to the question of whether changes in nuclear accumulation of MIER1α are related to the fact that the cells are proliferating. We therefore examined MIER1α localization in cells treated with E2, a classic mitogen for ER + breast carcinoma cells. Unlike insulin and IGF-1, E2 had no significant effect on nuclear accumulation of MIER1α (Fig. 4). In the presence of E2, 77 % of cells displayed nuclear MIER1α (Fig. 4Af, j, B) compared to 80 % of untreated cells (Fig. 4Ab, B). Likewise there was no significant difference in the percentage of cells with ‘whole cell’ or ‘cytoplasmic’ staining (Fig. 4B). These results demonstrate that the shift in MIER1α localization directed by insulin and IGF-1 is not the non-specific result of cell proliferation.

More than likely activation of one of the insulin/IGF signalling pathways is responsible for the altered MIER1α localization in MCF7. This type of effect has been observed for the FOXO family of transcription factors. For example, FOXO is driven out of the nucleus by insulin as well as other growth factors [13] and by Src signalling [14]. In *C. elegans*, activation of DAF-2, the nematode ortholog of the IGF-1 receptor, prevents nuclear accumulation of the DAF-16 (FOXO) transcription factor [15].

The data presented here demonstrate that for MCF7 the culture conditions, specifically the common practice of including insulin in the medium, can have important consequences for studies of nuclear proteins like MIER1α.

## Abbreviations

CBP: creb binding protein
CS: calf serum
E2: 17-β estradiol
ERα: estrogen receptor alpha
FBS: fetal bovine serum
HDAC: histone deacetylase
IGF-1: insulin-like growth factor 1
NLS: nuclear localization signal
